# Evaluation of Microhardness and Compressive Strength of Mineral Trioxide Aggregate Modified by Addition of Short Glass Fibers and Shredded Polyglycolic Acid Sutures

**DOI:** 10.3390/ma18071491

**Published:** 2025-03-26

**Authors:** Josip Filipović, Ana Ivanišević, Jurica Matijević, Ana Pilipović, Ivan Zajc, Ivana Miletić, Anja Baraba

**Affiliations:** 1 Health Center Vinkovci, Ulica Kralja Zvonimira 53, 32100 Vinkovci, Croatia; filipovic.dr@gmail.com; 2 University of Zagreb School of Dental Medicine, Department of Endodontics and Restorative Dentistry, Gundulićeva 5, 10000 Zagreb, Croatia; matijevic@sfzg.unizg.hr (J.M.); miletic@sfzg.unizg.hr (I.M.); 3 University of Zagreb Faculty of Mechanical Engineering and Naval Architecture, Lučićeva 5, 10000 Zagreb, Croatia; ana.pilipovic@fsb.unizg.hr; 4 University of Zagreb School of Dental Medicine, Department of Oral Surgery, Gundulićeva 5, 10000 Zagreb, Croatia; zajc@sfzg.unizg.hr

**Keywords:** compressive strength, glass fibers, microhardness, mineral trioxide aggregate, polyglycolic acid

## Abstract

The purpose of this study was to test the microhardness and compressive strength of mineral trioxide aggregate (MTA) modified by the addition of short glass fibers (SGFs) and shredded polyglycolic acid (PGA) sutures. Encapsulated MTA (MM-MTA, MicroMega, Besançon, France), modified using either SGF or shredded PGA sutures, was used for the experiment. Four experimental groups (n = 120) were as follows: control group (MTA) (n = 30), MM MTA + 5%SGF (n = 30), MM MTA + 10%SGF (n = 30), and MM MTA + 1%PGA (n = 30). For the modified materials, MM MTA powder was removed from the capsule by 1%, 5% and 10% of weight and 1% PGA, 5%, or 10% SGF were added, respectively. The microhardness of the samples (n = 20 per group) was measured using a Vickers microhardness testing machine, while compressive strength (n = 10 per group) was measured according to ISO 9917-1:2007. The highest microhardness value was measured for MTA + 10%SGF (14.73 ± 3.09) with a statistically significant difference in comparison to the other three groups (*p* < 0.05). Statistically significant higher compressive strength was measured in the groups with the addition of 5% and 10% SGF compared to MM MTA (*p* = 0.047 for both comparisons). There were no statistically significant differences between the groups (*p* = 0.784) regarding the compressive modulus. The addition of SGF significantly increased both the microhardness and compressive strength of MM MTA.

## 1. Introduction

Hydraulic calcium silicate cements (HCSCs) have been established as a valuable adjunct in various indications of endodontic therapy, particularly because of their bioactivity and biocompatibility, and are among most researched materials in dental medicine [1,2]. Mineral trioxide aggregate (MTA) is considered the golden standard, and as such, it has undergone several modifications to improve its color and color stability, simplify manipulation, which was challenging in earlier generations of the material, shorten the setting time, and enhance resistance to flushing out from the placement site.

Since MTA is used in both the apical part of the root, in the cases of root end resection procedures, and in the middle and coronal parts of the root as a repair material, these improvements affect clinical outcomes in different ways. In apical surgery or surgical root repair, the application of the material can be difficult due to the confined operating field, limited operative time and potential bleeding from surrounding tissues and bone. These factors may complicate the placement of the material, its setting properties, resistance to flushing out from the placement site, mechanical properties, and/or lead to marginal leakage microbial contamination and flare-ups [3].

In this context, ease of application, rapid setting, and moisture tolerance are desirable properties. For orthograde root or coronal repair, in addition to these challenges, there are also concerns about the long-term mechanical stability of the material, especially when subjected to occlusal and masticatory stresses, as well as temperature change challenges. Additionally, in the further reconstruction of the root or crown repaired with MTA, the material may experience significant mechanical and thermal stress [4]. These stresses can arise from root canal post and core preparation, cementation, or reconstruction using materials that undergo volume changes during setting or polymerization and have different thermo-mechanical properties compared to MTA [4,5].

Failure to withstand all these challenges may lead to the immediate failure of the repair or delayed clinical failure, resulting in the loss of the tooth and damage to surrounding tissues, which in turn can complicate further treatment. Therefore, increased mechanical stability, along with improved tolerance to thermo-mechanical stress, is needed, as well as rapid setting and reduced susceptibility to the influence of restorative materials with differing chemistries and mechanical behaviors [6,7].

Modifications of mineral trioxide aggregate (MTA) have been explored in terms of chemical composition, varying liquid-to-powder ratios, and the incorporation of bioglass, nanoparticles, organic polymers or glass fibers [6,8,9,10,11,12,13]. These modifications have focused on improving the material’s properties, such as its chemical, physical, and antibacterial characteristics [6,12,14,15,16].

Caprolactone-based compositions, modified with caffeic acid and added to MTA, have demonstrated promising osteogenic properties [17]. These compositions have shown potential in regulating vascular induction and osteogenic regeneration in dental pulp stem cells [17]. However, the mechanical properties of polyglycolic acid polymers have been found to be superior to those of caprolactone [18].

The addition of 5% alkaline-resistant glass fibers has been shown to increase the fracture resistance of both MTA and Biodentine [10]. While E-type glass fibers are less resistant to alkaline environments than their alkaline-resistant counterparts, they have proven effective in the production of various dental composites, significantly improving their mechanical properties [19]. According to the available literature, there are no studies on the effect of incorporating E- type SGF or PGA into MTA.

The aim of this study is to evaluate changes in the mechanical properties, microhardness, and the compressive strength, of the commonly used MTA material by adding different amounts of short glass fibers (SGFs) and shredded polyglycolic acid (PGA) sutures. The null hypothesis was that the addition of SGF or PGA would not affect the mechanical properties of the MTA material.

## 2. Materials and Methods

Encapsulated MTA (MM MTA, MicroMega, Besançon, France), modified using either SGF, with a length of 140 µm and diameter 6 µm (Central Glass Co., Tokyo, Japan) or shredded PGA sutures, size 3/0 (Pegesorb, Dogsan Inc., Trabzon, Turkey), was used for the experiment. Four experimental groups (n = 120) were as follows: control group (MM MTA) (n = 30), MM MTA + 5%SGF (n = 30), MM MTA + 10%SGF (n = 30), and MM MTA + 1%PGA (n = 30). For the modified materials, MM MTA powder was removed from the capsule by 1%, 5%, and 10% of weight and 1% PGA, 5% or 10% SGF were added, respectively. PGA was first shredded in a blender and then further ground in a mortar with a pestle. When added to the capsule, SGF and PGA were mixed with MTA powder using a Heidemann instrument before capsule mixing, until a homogeneous distribution of PGA in the MTA powder was achieved. The materials were mixed according to the manufacturer’s instructions and placed in custom made Teflon molds, 6 mm in diameter and 4 mm in height, for measurement of microhardness (n = 20 per group). After seven days of incubation in phosphate-buffered saline (PBS), the samples in Teflon molds were polished using sandpaper, from coarse to fine, in a polishing device (Minitech 250, Presi, Eybens, France). Microhardness of samples was measured using a Vickers microhardness testing machine (KB Prüftechnik GmbH, Hochdorf-Assenheim, Germany). A pyramid-shaped diamond peak was used to apply pressure of 200 g for 10 s. Three indents were made on each tested sample, and the mean Vickers hardness values were calculated and expressed in HV.

Samples for compressive strength testing were prepared using custom made Teflon molds 3 mm in diameter and 4 mm in height (n = 10 per group). After seven days of incubation of the samples in PBS, compressive strength was measured according to ISO 9917-1:2007, at a speed of 0.75 mm/min, at room temperature (~22 °C) and relative humidity of 45%. Load was applied along the long axis of the specimen, while the specimen was placed with its flat ends between the plates of the testing machine (Shimadzu AGS-X, Shimadzu, Kyoto, Japan), with a maximal force of 10 kN. The maximum load required to fracture each specimen was recorded and the compressive strength was eventually determined from the maximum load and the surface area of the specimens according to the following equation:

$$C=\frac{4\cdot F}{\pi \cdot d^{2}}$$

where *C* [N/mm^2^] is compressive strength, *F* [N] is maximum force, and *d* [mm] is diameter of the test specimen.

Compressive modulus was calculated according to the following equation:

$$E=\frac{\sigma }{\varepsilon }=\frac{\sigma_{2}-\sigma_{1}}{\varepsilon_{2}-\varepsilon_{1}}$$

where *σ*, *σ*_1_, *σ*_2_ [N/mm^2^] is strength, *ε*, *ε*_1_, *ε*_2_ [%] is strain. In the case of these tests, the values of strain *ε*_1_, *ε*_2_ was taken for *σ*_1_ = 0.5 N/mm^2^, *σ*_2_ = 1.5 N/mm^2^.

The results for compressive strength and modulus were intended to be presented as mean values in the table and depicted in the stress–strain diagram.

For qualitative SEM analysis, one sample from each group was embedded into a polymer-based electrically conductive mass and pressed (Mecapress 3, Pressi, Eybens, France). The samples were then coarsely and finely ground using an automatic sample preparation device (Mecatech 250, Pressi, Eybens, France) and observed under SEM (Axia ChemiSEM, ThermoFisher Scientific, Seattle, WA, USA), at magnifications of 20× and 500×.

For statistical analysis of microhardness, the Kolmogorov–Smirnov normality test and Student’s *t*-test for independent samples were applied. For compressive strength, differences between groups were analyzed using a non-parametric Kruskal–Wallis analysis of variance, and individual differences between groups were also further tested using the same analysis. The analysis was performed using IBM SPSS software, version 21. The level of statistical significance was set at 5%.

## 3. Results

### 3.1. Microhardness and Compressive Strength

The highest microhardness value was measured for MM MTA + 10% SGF (14.73 ± 3.09), with statistically significant difference compared to other three groups (*p* = 0.001). Both MTA + 5%SGF and MTA + 1%PGA microhardness values (12.10 ± 2.44 and 10.77 ± 2.70, respectively) were statistically significantly different from the control group (7.76 ± 3.09), (*p* = 0.001 and *p* = 0.002, respectively). There was no statistically significant difference in microhardness of MM MTA + 5%SGF and MM MTA + 1%PGA (*p* = 0.11).

**Figure 1:**
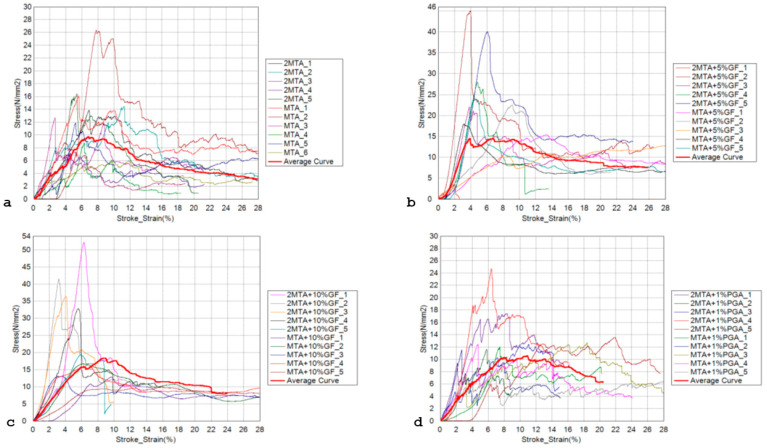
Compressive properties: stress–strain curves for (**a**) MM MTA; (**b**) MM MTA + 5%SGF; (**c**) MM MTA + 10%SGF; and (**d**) MM MTA + 1%PGA.

Compressive stress–strain curves are presented in Figure 1. The individual curves for each test specimen are shown in the diagram, with the mean value curves highlighted (marked with a bold red line) (Figure 1). The mean value curves were determined using the least squares method. For each individual test specimen, the universal testing machine provided force and displacement data every 0.01 s. From these data, the stresses and strains were calculated. Then, the stress and strain data from each test specimen were processed using the least squares method to calculate the individual points, and these points were used to draw the curves. From these curves, data for compressive strength and deformation can be extracted, and the compressive modulus can be calculated according to the equation and the slope of the curve relative to the x axis (Figure 1). The results for compressive strength showed statistically significant differences between the groups (*p* = 0.006). When comparing individual groups, statistically significantly higher compressive strength was measured in the groups with the addition of 5% and 10% SGF compared to MM MTA (*p* = 0.047 for both comparisons), while there were no statistically significant differences between other pairs of groups (Table 1). Regarding the compressive modulus, the differences between the groups were not statistically significant (*p* = 0.784), (Table 1).

### 3.2. SEM Analysis

**Figure 2:**
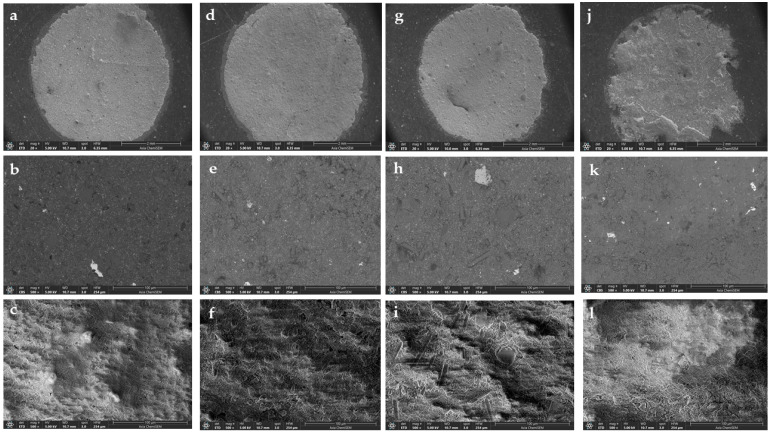
SEM images of MM MTA under (**a**) low (20×, ETD); (**b**,**c**) high (500×, CBS and ETD) magnification; MM MTA + 5%SGF under: (**d**) low (20×, ETD); (**e**,**f**) high (500×, CBS and ETD) magnification; MM MTA + 10%SGF under: (**g**) low (20×, ETD); (**h**,**i**) high (500×, CBS and ETD) magnification; MM MTA + 1%PGA under: (**j**) low (20×, ETD); (**k**,**l**) high (500×, CBS and ETD) magnification.

SEM imaging was performed under low (20×) and high (500×) magnification (Figure 2). At higher magnification, two detectors were used: Everhardt–Thornley detector (ETD) for secondary electrons and the concentric back-scattered electron detector (CBS), which provided insights into sub-surface structures (Figure 2b,c,e,f,h,i,k,l). At low magnification, only ETD was used (Figure 2a,d,g,j). Low magnification images did not reveal any significant differences between MM MTA (control), MM MTA + 5%SGF, and MM MTA + 10%SGF groups (Figure 2a,d,g). However, MM MTA + 1%PGA showed increased surface irregularities (Figure 2j).

At high magnification, the MM MTA + 10%SGF group clearly displayed glass fibers in the material, especially in CBS mode (Figure 2h,i). In the MM MTA + 5%SGF group, identifying the fibers, was more challenging, especially in ETD mode (Figure 2e,f). Surface morphology between these two groups was comparable in ETD mode (Figure 2f,i). In contrast, the MM MTA + 1%PGA group exhibited distinct surface morphology with more pronounced irregularities (ETD mode), (Figure 2l). PGA fibers were difficult to observe in CBS mode (Figure 2k), and the surface irregularities were most evident in ETD mode (Figure 2l). Crystalization patterns were also detected in the MM MTA + 1%PGA group (Figure 2l).

## 4. Discussion

This study examined the microhardness and compressive strength of MM MTA modified by the addition of SGF and PGA.

Before selecting the percentages of SGF and PGA to be added to MM MTA for the study, different percentages of both SGF and PGA, in ascending order, were added to MM MTA to assess whether the material could be properly mixed and extruded from the capsule. However, it was found that, after adding more than 10% of SGF (e.g., 15%), the material could not be extruded from the capsule after mixing according to the manufacturer’s instructions. This issue also occurred with the addition of only 2% of PGA. Therefore, the final decision was to add 5% and 10% of SGF and 1% of PGA to MM MTA for testing the mechanical properties of the modified materials.

In the present study, the addition of SGF to MTA increased its compressive strength and microhardness, with the increase being more pronounced with the addition of 10 wt% SGF. The addition of 1 wt% shredded PGA to MTA had no significant effect on compressive strength, but microhardness was significantly higher. The compressive modulus was not significantly affected by the addition of SGF or PGA. The null hypotheses that the addition of SGF and PGA would not influence microhardness and compressive strength was rejected. The compressive modulus was not influenced by the addition of PGA and SGF to MTA, indicating that the material’s stiffness was not reduced after modifications.

Considering the clinical procedures in which the use of HCSCs is indicated, mechanical properties do not appear to be crucial for clinical success, since the material is only indirectly exposed to masticatory forces [14]. However, it has been shown that the improved mechanical properties of HCSCs, when used as sealers or intra-orifice barrier materials, have a positive impact on the root fracture resistance [10,20].

Although the sealing of MTA was effective when used as an intra-orifice barrier [21,22], its poor mechanical properties were shown to negatively affect root fracture resistance when the material was used as intra-orifice barrier [23]. In this context, compressive strength is the most relevant property, since most masticatory forces are compressive in nature. Additionally, the microhardness of the material defines its resilience when placed under permanent restoration, or when it is used for repairing furcal perforations or pulp capping [8,9].

Compressive strength testing according to ISO9917-1 involves placing samples in molds for only one hour prior to testing [24,25]. However, it takes longer for MTA and other HCSCs to set, and they require a moist environment for proper setting [14]. That is why tests in previous studies were performed after several days or weeks, which is not in accordance with ISO 9917, and it was shown that the compressive strength values increased with time [8,26,27]. Since the aim of the present study was not to test the change in the mechanical properties of the modified materials over time, but rather to assess the impact of the modification on the mechanical properties, the tests were performed at a single time point. In accordance with ISO 9917 and considering the prolonged curing of MTA, the commercial and modified materials were tested after 7 days, when the materials were fully cured, although maturation was not yet complete [12,25]. In future investigations, it would be interesting to examine how compressive strength and microhardness change over time, and whether the improvement in mechanical properties inherent in MTA [12] is also observed in materials modified with PGA and SGF.

Furthermore, the storage medium significantly affected physical properties of HCSCs [26,28]. Compressive strength was higher and was solubility lower when samples were stored in water compared to other storage media containing ions, glucose and proteins [26,27,28]. Water is not a suitable storage medium for HCSCs in in vitro studies because, in in vivo conditions, hydraulic cements interact with ions and proteins in tissue fluids, which alter their properties [28]. In fact, it has been suggested that for mechanical properties testing, MTA should be submerged in PBS, (wet cured), and tested after at least 24 h, and at other points in time, e.g., 7, 21, or 28 days [25].

This may explain why the absolute values of compressive strength and microhardness obtained in the present study are lower than the previously reported values for the commercially available MTA cements and other HCSCs, as the samples in the present study were stored in PBS [8,9,10,26,27].

The absolute values of compressive strength and microhardness obtained in the present study are lower than the literature data for commercially available materials; however, the comparisons between the groups in the present study provide relevant information on the effect of adding 5 wt% and 10 wt% SGF and 1 wt% PGA to a commercial MTA material. According to the available literature, there is no study on the effect of adding PGA to HCSCs. PGA is a biodegradable linear aliphatic polyester, used to produce absorbable surgical sutures for decades [29]. When exposed to extracellular fluid, PGA is degraded to glycolic acid by random hydrolysis and by the action of tissue esterase. When used as absorbable suture, PGA loses its strength after four weeks, which is within the time frame during which HCSCs completely sets, and it is completely absorbed in two to three months [25,30].

It the present study, MTA modified with PGA shredded sutures did not result in significantly improved compressive strength, but microhardness was significantly improved. This may be clinically relevant if MTA is used for the repair of furcation perforations, where masticatory forces are indirectly exerted on the HCSCs cement and its resilience is desirable. One side of the repair material is exposed to extracellular fluid/blood. It was shown that blood interferes with the hydration process and lowers the microhardness of MTA [28]. The addition of PGA may help compensate for this lowering of microhardness due to the exposure to blood/extracellular fluid. Nevertheless, exposure to extracellular fluid promotes the hydrolytic degradation of PGA and hydration of HCSCs with the deposition of carbonated apatite [31]. It may also be expected that the cells would adhere and proliferate on the surface of a bioactive material such as MTA modified with PGA, potentially covering the gaps and notches on the surface created after the degradation of exposed PGA sutures on the surface of PGA-modified MTA [31].

As already mentioned, compressive strength was not affected by the addition of PGA. This may be because there were no bonds between the shredded PGA fibers and the cement matrix, so there could not be a sufficient stress transfer from the cement matrix to the fibers.

Glycolic acid, the main component of PGA, has been reported as a hydration inhibitor for tricalcium silicates by interfering with calcium hydroxide precipitation during hydration [32]. Glycolic acid is a hydroxycarboxylic acid with two hydroxyl groups and one carboxyl group. In chelation, the hydroxyl groups of the carboxylic acid molecules can lose a proton and coordinate with a Ca^2+^ ion, forming calcium glycolate. Calcium glycolate forms a strong hydrogen bonding network near Ca(OH)_2_ and hydrated tricalcium silicate surfaces, strongly adsorbing to these surfaces [33]. Since the hydration of tricalcium silicate is responsible for early strength development in Portland cement [33], the incorporation of PGA fibers may have affected the rate of hydration and influenced the compressive strength results of MTA observed in the present study. Further research could explore the effect of PGA on cement hydration using techniques such as colorimetry experiments, X-ray diffraction, adsorption experiments, and molecular modeling [33,34].

However, strengthening was observed in the experimental groups modified with SGF. It is known that the addition of SGF to dental materials improves their mechanical properties without compromising their biocompatibility [35]. It has been previously shown that the incorporation of 5 wt% SGF into HCSCs resulted in better diametral tensile strength while compressive strength was comparable for 5 and 10 wt% SGF [10]. The results of Nagas et al. [10] are in accordance with the results of the present study, where both weight percentages significantly improved compressive strength without statistically significant difference between two groups.

Moreover, in the present study, the modification with 10 wt% SGF resulted in superior microhardness, which was not tested in previous studies. The difference may be explained by the fact that the optimization of matrix/fiber ratio is influenced by the size of the fibers. The length of the fibers in the present study was shorter, and diameter smaller (140 µm and 6 µm, respectively) compared to the fibers in the Nagas et al. [10] study (1 mm and 14 µm, respectively). Additionally, Nagas et al. [10] used AR fibers containing zirconium, which are resistant to partial degradation in the alkaline environment, resulting in a weaker bond between the fibers and HCSCs. MTA and SGF components are compatible and enable chemical bonding. MTA contains tricalcium silicate, dicalcium silicate, tricalcium aluminate, and tetracalcium aluminoferrite. The glass fibers used in this research are non-alkali glass (E-type). E-type or alumino-borosilicate glass is a mixture of SiO_2_, Al_2_O_3_, B_2_O_3_, CaO, MgO, and <1% of alkali oxides (Na_2_O, K_2_O). Due to its low alkali metal oxide content, borosilicate glass has high hydrolytic resistance. Also, high wt% of SiO_2_ (52–56%) ensures high acid resistance, but it is only moderately resistant to alkaline solutions. During the initial hydration in HCSCs, C-S-H gel and Ca(OH)_2_ are formed, and, after Ca(OH)_2_ dissociates, pH rises [31]. This alkaline environment is responsible for the partial degradation of E-type glass fibers during the setting of the cement, because it can be assumed that OH⁻ ions attack the surfaces of SGF, causing partial hydrolysis, ion leaching, and the formation of an amorphous silicate hydrate gel, similar to the amorphous calcium silicate hydrate gel formed in the HCSCs cement. This non-stoichiometric gel layer contains silanol (Si–OH) groups, which facilitate bonding between the cement and fibers through these silanol groups. Future investigations, including FTIR analysis, should be conducted to characterize the chemical interactions between SGF and HCSCs, and potentially confirm the proposed interactions.

The mechanical properties of the materials tested do not entirely reflect their clinical performance, especially considering the interaction between the material with the tissues. The in vitro testing of mechanical properties enables comparisons between the original material and experimental formulations, and supports the modification of MTA with 10 wt% SGF in clinical situations where masticatory forces are more pronounced. Although the results of this study indicate that the addition of 10 wt% SGF to MTA enhanced its properties, further studies are needed to better understand the mechanism behind the improved mechanical performance. Shear rheology experiments could provide additional insights into the varying degrees of deformation, depending on the strength of the interaction between the additive and the cement matrix. Additional research should also be conducted to evaluate the interaction between the new formulations with tissues and to test their biocompatibility.

## 5. Conclusions

Within the limitations of the current study and using PBS as storage medium, the addition of SGF or shredded PGA sutures significantly increased the microhardness of MM MTA. However, the highest microhardness values were obtained by adding 10% of SGF to MM MTA. The addition of both 5% and 10% SGF was found to increase the compressive strength of MM MTA, while adding 1% PGA did not increase the compressive strength of the material. The addition of 10% SGF could enhance the mechanical properties and endurance of MM MTA. Despite the results obtained in the present study, further research is needed to evaluate both the mechanical properties and clinical behavior of experimental MTA.

## Figures and Tables

**Table 1:** Descriptive statistics of compressive strength and compressive modulus. Different letters in superscript indicate statistically significant differences between the groups.

			Compressive Strength [MPa]	Compressive Modulus [MPa]
**Material**	**MM MTA**	Mean	11.594 ^a^	279.254 ^a^
		Standard deviation	3.731	168.343
		Minimum	5.927	115.069
		Percentile 25	8.737	152.237
		Median	12.250	203.753
		Percentile 75	14.428	423.411
		Maximum	16.409	563.189
	**MM MTA + 5% SGF**	Mean	23.189 ^b^	249.857 ^a^
		Standard deviation	12.317	114.570
		Minimum	3.569	125.323
		Percentile 25	15.355	168.728
		Median	22.698	245.659
		Percentile 75	27.927	280.553
		Maximum	45.005	474.254
	**MM MTA + 10% SGF**	Mean	25.149 ^b^	286.228 ^a^
		Standard deviation	14.540	137.843
		Minimum	9.884	117.869
		Percentile 25	13.104	165.564
		Median	18.039	272.778
		Percentile 75	36.803	344.381
		Maximum	52.197	536.189
	**MM MTA + 1% PGA**	Mean	13.547 ^a^	215.058 ^a^
		Standard deviation	4.639	88.883
		Minimum	7.085	67.123
		Percentile 25	11.602	144.983
		Median	12.450	205.464
		Percentile 75	13.552	289.389
		Maximum	24.666	337.280

## Data Availability

The original contributions presented in this study are included in the article. Further inquiries can be directed to the corresponding authors.
